# Real-world adherence and persistence for newly-prescribed HIV treatment: single versus multiple tablet regimen comparison among US medicaid beneficiaries

**DOI:** 10.1186/s12981-020-00268-1

**Published:** 2020-04-01

**Authors:** Joshua Cohen, Anne Beaubrun, Richa Bashyal, Ahong Huang, Jieni Li, Onur Baser

**Affiliations:** 1 Tufts University, Boston, MA USA; 2 Gilead Sciences Inc, Foster City, CA USA; 3 STATinMED Research, Plano, TX USA; 4 Columbia University, New York, NY USA; 5 University of Michigan, Ann Arbor, MI USA

**Keywords:** HIV, Antiretroviral therapy, Adherence, Persistence, Discontinuation

## Abstract

**Background:**

Once-daily, single-tablet regimens (STRs) have been associated with improved patient outcomes compared to multi-tablet regimens (MTRs). This study evaluated real world adherence and persistence of HIV antiretroviral therapy (ART), comparing STRs and MTRs.

**Methods:**

Adult Medicaid beneficiaries (aged ≥ 18 years) initiating ART with ≥ 2 ART claims during the identification period (January 1, 2015–December 31, 2016) and continuous health plan enrollment for a 12-month baseline period were included. For STRs, the first ART claim date was defined as the index date; for MTRs, the prescription fill claim date for the last drug in the regimen was defined as the index date, and prescription fills were required to occur within a 5-day window. Adherence was assessed in 30-day intervals over a 6-month period, with adherence defined as having less than a 5-day gap between fills. Persistence was evaluated as median number of days on therapy and percent persistence at 12 months. Cox Proportional Hazard models were used to evaluate risk of discontinuation, controlling for baseline and clinical characteristics.

**Results:**

A total of 1,744 (STR = 1290; MTR = 454) and 2409 (STR = 1782; MTR = 627) patients newly prescribed ART had available data concerning adherence and persistence, respectively. Average age ranged 40–42 years. The patient population was predominantly male. Adherence assessments showed 22.7% of STR initiators were adherent to their index regimens over a 6-month period compared to 11.7% of MTR initiators. Unadjusted persistence analysis showed 36.3% of STR initiators discontinued first-line therapy compared to 48.8% for MTR initiators over the 2-year study period. Controlling for baseline demographic and clinical characteristics, MTR initiators had a higher risk of treatment discontinuation (hazard ratio [HR] = 1.6, p < 0.0001). Among STRs, compared to the referent elvitegravir(EVG)/cobicistat(COBI)/emtricitabine(FTC)/tenofovir alafenamide(TAF), risk of discontinuation was higher for efavirenz(EFV)/FTC/tenofovir disoproxil fumarate(TDF) (HR = 3.6, p < 0.0001), EVG/COBI/FTC/TDF (HR = 2.8, p < 0.0001), and abacavir (ABC)/lamivudine (3TC)/dolutegravir (DTG) (HR = 1.8, p = 0.004). Among backbones, FTC/TAF was associated with lower risk of discontinuation than FTC/TDF (HR = 4.4, p < 0.0001) and ABC/3TC (HR = 2.2, p < 0.0001).

**Conclusions:**

Among patients newly prescribed ART, STR initiators were significantly less likely to discontinue therapy and had greater adherence and persistence compared to MTR initiators. Regimens containing FTC/TAF as a backbone had higher persistence than those consisting of other backbones.

## Background

At the end of 2015, ~ 1.1 million people aged ≥ 13 years were living with human immunodeficiency virus (HIV) in the United States [1]. While estimated annual HIV infections in the United States have declined by 10% from 2010 to 2014 [1], the condition remains a serious public health concern given the burden it imposes on patients and the health care system.

HIV treatment typically includes a combination of antiretroviral therapy (ART) regimens to prevent HIV disease progression and transmission [2]. ART has brought about a substantial decrease in mortality due to HIV infection, changing it from a rapidly lethal disease into a chronic manageable condition [3]. In fact, the use of ART has significantly shown an improvement in the life-expectancy and quality of life (QoL) among people infected with HIV [4, 5]. Moreover, early initiation of ART has shown improvement in clinical outcomes and reduction in sexual transmission of HIV through viral suppression [5]. The Department of Health and Human Services (DHHS) panel on antiretroviral guidelines for adults and adolescents also recommends immediate initiation of ART for all people living with HIV, regardless of CD4 count, to reduce the morbidity and mortality associated with HIV infection [6]. These guidelines recommend initiating ART in treatment naïve patients with a regimen consisting of two nucleoside reverse transcriptase inhibitors (NRTIs) along with a third drug: preferably an integrase strand transfer inhibitor (INSTI), with non-nucleoside reverse transcriptase inhibitors (NNRTI) or boosted protease inhibitors (PI) as alternatives to INSTIs [5–7].

Combination ART was initially characterized by high pill burden and multiple daily doses [8–10]. Over the past two decades, more potent, more convenient, and less-toxic ART regimens have been developed; treatment regimens of up to 20 pills per day have been largely replaced by once-daily, single-tablet regimens (STRs) [10, 11]. As of March 2016, six STRs—elvitegravir (EVG)/cobicistat (COBI)/emtricitabine (FTC)/tenofovir alafenamide fumarate (TAF), EVG/COBI/FTC/tenofovir disoproxil fumarate (TDF), dolutegravir (DTG)/abacavir (ABC)//lamivudine (3TC), FTC/rilpivirine (RPV)/TAF, FTC/RPV/TDF, and efavirenz (EFV)/FTC/TDF—had been approved by the US Food and Drug Administration for HIV treatment [12]. STRs are single-dose units administered once-daily, whereas multi-tablet regimens (MTRs) require multiple dosing units per day [4]. STRs may improve adherence, thus resulting in greater efficacy, reduced risk of virologic failure, and prevention of the emergence of drug resistance [13, 14]. The decision to prescribe STRs vs MTRs is based to a large extent on patient and clinician preference as well as, in some instances, medication cost and positioning of products on payer formularies [15, 16]. Moreover, HIV infection requires a high level of adherence to achieve viral suppression, presenting a challenge for HIV management, particularly among patients who face barriers such as limited provider and pharmacy services, low social support, and substance use [17, 18]. Reducing pill burden and structural barriers may therefore help patients achieve increased adherence and improved health outcomes.

Prior research suggests that adherence and persistence may be higher among patients treated with STRs compared with those treated with MTRs [13, 19]. However, with the advent of new treatment options with greater effectiveness and tolerability, the extent to which adherence and persistence varies between and within STRs and MTRs has not been fully investigated. Amongst third agents, INSTIs are the most preferred as evident in studies that showed it be superior or equivalent to other third agents in safety and efficiency. In addition, it is reported that INSTIs are more tolerable to patients, in turn reducing discontinuation rates [20–23]. However, for patients that are at higher risk of non-adherence, PIs as the third agent would be most appropriate because of their genetic barrier to resistance [24]. For backbones, tenofovir-containing nucleoside has shown higher safety and tolerability then abacavir-lamivudine [25]. In addition, tenofovir/emtricitabine has been found to have a lower rate of discontinuation than co-formulated abacavir/lamivudine [20]. Hence, this retrospective claims-based study was conducted with an aim to assess real world adherence and persistence for newly prescribed HIV treatment comparing STRs versus MTRs, backbones, and third agents using the Truven Health Medicaid database.

## Methods

### Data source

This retrospective real-world study utilized data from the Truven Health Medicaid database from January 1, 2014 through December 31, 2016 (study period), with an approximately 6 months lag time. The Truven Health Medicaid Database contains medical, surgical, and prescription drug claims for more than 44 million Medicaid enrollees from multiple states. The database includes records of inpatient services, inpatient admissions, outpatient services, and prescription drug claims as well as information about long-term and other medical care. Data on eligibility (by month), service, and provider type are also included. In addition to standard demographic variables including age, gender, and race, the database also included variables more specific for Medicaid populations including aid category (e.g., blind or disabled, medicare eligible).

### Study population

Adult patients (aged ≥ 18 years) initiating ART after one pre-index year of not receiving ART (i.e. presumably beginning first-line ART for the majority of patients) with ≥ 2 claims for an ART of interest (Table 1) during the identification period (January 1, 2015 through December 31, 2016) were included. For STRs, the first ART claim date during the identification period was designated as the index date. For MTRs, the prescription fill claim date of the last drug in the regimen was identified as the index date. MTRs consisting of two drug claims, both therapies in the regimen, were required to have been filled within a 5-day window. For MTRs with a boosted agent (ritonavir or COBI), the boosted agent was required to have been filled within 5 days of the second drug in the regimen. For example, for an FTC/TDF + darunavir (DRV) regimen boosted with COBI, the DRV prescription was required to have been filled within 5 days of FTC/TDF, and the COBI prescription was filled within 5 days of DRV. Patients prescribed any other ART outside of the common, guideline-recommended ARTs of interest, or those with an inappropriate MTR regimen, were excluded from the study. Eligible patients had continuous health plan enrollment in the Truven Medicaid population for a 12-month baseline period. If patients lost Medicaid coverage within 6 months of follow-up, they were excluded from the study. Patients were selected based on two enrollment criteria: (1) assess adherence among patients with ≥ 6-month follow-up period, and (2) assess persistence among patients with a follow-up period through the earlier of either the end of continuous enrollment or end of the study period. Patients who died during the follow-up period were not included in the study. Eligible patients were further stratified into STR and MTR cohorts based on their index prescription claim. STR and MTR initiators were further sub-stratified based on the individual STR drug and MTR regimen.

**Table 1:** Antiretroviral therapies of interest

STR		MTR	
Brand name	Components	Brand name (Regimen)	Components
Genvoya^®^	EVG/COBI/FTC/TAF	Epzicom^®^ + Prezista^®^ boosted with ritonavir	ABC/3TC + DRV/r
Stribild^®^	EVG/COBI/FTC/TDF	Epzicom^®^ + Prezista^®^ boosted with COBI	ABC/3TC + DRV/c
Triumeq^®^	ABC/3TC/DTG	Truvada^®^ + Tivicay^®^	FTC/TDF + DTG
Odefsey^®^	RPV/FTC/TAF	Descovy^®^ + Tivicay^®^	FTC/TAF + DTG
Complera^®^	RPV/FTC/TDF	Truvada^®^ + Prezista^®^ boosted with ritonavir	FTC/TDF + DRV/r
Atripla^®^	EFV/FTC/TDF	Truvada^®^ + Prezista^®^ boosted with COBI	FTC/TDF + DRV/c
		Descovy^®^ + Prezista^®^ boosted with ritonavir	FTC/TAF + DRV/r
		Descovy^®^ + Prezista^®^ boosted with COBI	FTC/TAF + DRV/c
		Truvada^®^ + Reyataz^®^ boosted with ritonavir	FTC/TDF + ATV/r
		Truvada^®^ + Reyataz^®^ boosted with COBI	FTC/TDF + ATV/c
		Descovy^®^ + Reyataz^®^ boosted with ritonavir	FTC/TAF + ATV/r
		Descovy^®^ + Reyataz^®^ boosted with COBI	FTC/TAF + ATV/c

*3TC* lamivudine, *ABC* abacavir, *ATV* atazanavir, *ATV/c* atazanavir boosted with cobicistat, *ATV/r* atazanavir boosted with ritonavir, *COBI* cobicistat, *DRV* darunavir, *DRV/r* darunavir boosted with ritonavir, *DRV/c* darunavir boosted with cobicistat, *DTG* dolutegravir, *EFV* efavirenz, *EVG* elvitegravir, *FTC* emtricitabine, *RPV* rilpivirine, *TAF* tenofovir alafenamide fumarate, *TDF* tenofovir disoproxil fumarate

### Demographic and baseline clinical characteristics

Patient demographics including age, gender, race, and insurance type were assessed. Additionally, clinical characteristics including pre-index medication use (i.e., antihypertensive, antidiabetics, anticoagulants, antiarrhythmic drugs, lipid-lowering therapy, antibiotics, and respiratory drugs for lower and upper respiratory infections), number of unique medications on index date except ART, Deyo-modified Charlson comorbidity index (CCI) score, and baseline individual comorbidities (i.e., central nervous system toxicity, gastrointestinal symptoms, mental disorders, AIDS-defining condition, substance abuse, jaundice, dyslipidemia, diabetes, chronic kidney disease, cardiovascular disease, and myocardial infarction; recognized using International Classification of Diseases, 9th Revision, Clinical Modification [ICD-9-CM] codes) were assessed. All ICD-9-CM codes were mapped to ICD-10-CM codes based on the General Equivalence Mappings published by the Centers for Medicare & Medicaid Services (CMS) [26].

### Outcome measures

Outcome measures, including adherence and persistence during the follow-up period were measured. In HIV, a high level of adherence to ART is required for viral suppression [13]. However, thresholds for categorizing “adherent” versus “non-adherent” differ across studies (e.g. taking 80% of prescribed [27], to taking 95%−100% of prescribed [28, 29]), and studies further differ in measures used for assessing adherence (e.g. self-report, proportion of days covered) and study time periods over which adherence is measured. In this study, adherence to first-line STRs and MTRs was categorized as adherent/non-adherent over the 6-month follow-up period, and was also assessed on a month-by-month basis (30-day intervals). Based on prior adherence measures assessing missed doses over 4−7 day periods [29–31], adherence was defined as ≤ 5-day gap between successive fills for patients initiating STRs, while non-adherence was defined as a > 5-day gap between successive fills, measured from the end of days’ supply of one fill and the claim date of the following fill during the 6-month follow-up period. Among patients who initiated an MTR, adherence was defined as ≤ 5 days in which one or more drugs in the regimen were not on hand according to prescription days of supply; non-adherence was defined as > 5 days in which one or more drugs in the regimen was not on hand. Among non-adherent patients, the refill gap (days) and proportion of patients with a cumulative refill gap was also calculated. Sensitivity analyses were also performed by using different gap thresholds (≤ 7 and ≤ 14 days) to evaluate the impact of expanding the gap window on adherence to STR.

Persistence for HIV treatments was compared between STRs versus MTRs, backbones, and third agents where persistence was measured from the index regimen start date until the start of the first 90-day gap between prescription fills or the end of the enrollment. Patients were considered to have discontinued first-line therapy if a gap of ≥ 90 days between fills was observed. Patients who restarted therapy after a ≥ 90-day gap remained classified as discontinued, as restarts were not captured in the analysis. MTR initiators were required to remain on all therapies in the initial regimen to be considered persistent. Among MTR initiators, a gap of ≥ 90 days for any drug in the regimen was considered discontinuation of that regimen. The proportion of patients who discontinued their first-line therapy, time to discontinuation, and the proportion of patients with 12 months of persistence were also measured. Additionally, persistence was assessed as the risk of treatment discontinuation among different treatment cohorts.

### Statistical analysis

All study variables, including baseline demographics and clinical characteristics, as well as outcome variables (i.e., adherence and persistence) were first examined descriptively. For continuous variables such as persistence measures, mean, median, and standard deviations were generated. For categorical variables, counts (frequencies) and percentages were reported.

Overall time to discontinuation of first-line ART for STRs and MTRs was assessed via Kaplan–Meier curves. Log-rank tests were used to evaluate statistical differences between treatment discontinuation curves. To assess the factors associated with risk of treatment discontinuation for each regimen, a multivariable Cox proportional hazard model was constructed. Covariates included in the multivariable-adjusted model comprised age group, gender, race, insurance type, CCI score, baseline individual comorbidities (more specific than CCI comorbidities), pre-index medication use, and the number of unique medications on index date. All of the analyses were conducted using SAS^®^ statistical software (Version 9.3, SAS Institute, Cary, North Carolina, 2012).

## Results

**Fig. 1:**
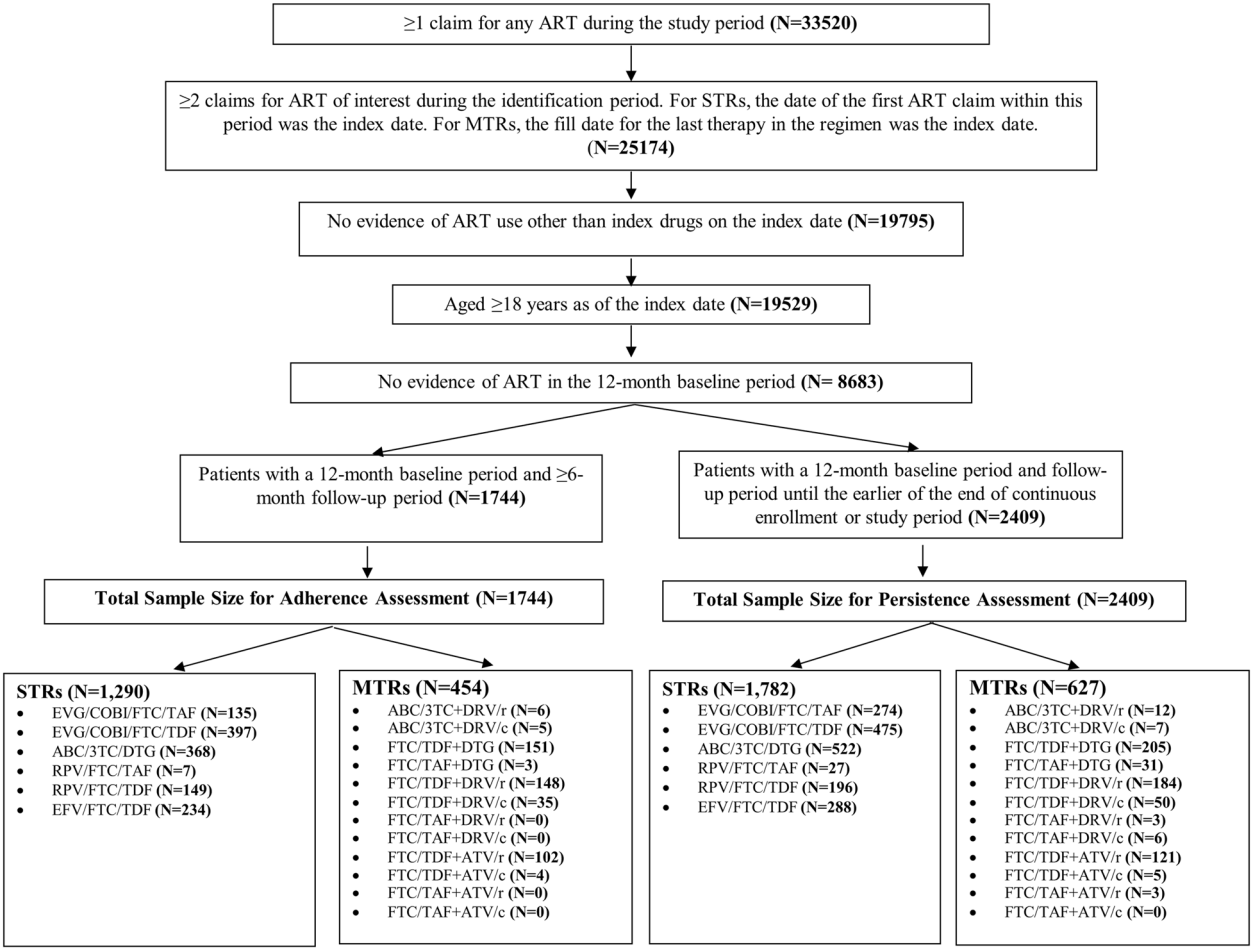
Study population and cohorts. *3TC* lamivudine, *ABC* abacavir, *ATV* atazanavir, *ATV/c* atazanavir boosted with cobicistat, *ATV/r* atazanavir boosted with ritonavir, *COBI* cobicistat, *DRV* darunavir, *DRV/r* darunavir boosted with ritonavir, *DRV/c*: darunavir boosted with cobicistat, *DTG*: dolutegravir; *EFV* efavirenz, *EVG* elvitegravir, *FTC* emtricitabine, *RPV* rilpivirine, *TAF* tenofovir alafenamide fumarate, *TDF* tenofovir disoproxil fumarate

For the adherence study, 1744 patients met inclusion criteria, of whom 1290 (74.0%) initiated STRs and 454 (26%) initiated MTRs. For the persistence study, 2409 patients met inclusion criteria, of whom 1782 (74.0%) initiated STRs and 627 (26.0%) initiated MTRs. Patients on STRs and MTRs were further stratified by specific ARTs to compare persistence within STRs and within MTRs (Fig. 1).

### Patient population characteristics

The average age of newly treated HIV patients was 40 (persistence sample) to 41 (adherence sample). The adherence study included 1744 patients, most (71.4%) of whom were aged 18–49 years, male (56.5%), black (61.0%), and were enrolled in a comprehensive insurance plan (74.4%). Among patients in the adherence sample, pre-index medication use was highest for respiratory drugs (54.9%) and antibiotics (43.5%); the most common comorbidities at baseline were mental health disorders (56.5%), substance abuse (41.5%), and central nervous system toxicity (40.9%). Mean CCI score during the baseline period was 4.2 ± 3.4. Patients taking STRs and MTRs had similar demographic and clinical characteristics (Table 2). Among the 2409 patients included in the persistence analysis, demographic and clinical characteristics were similar to patients included in the adherence cohort (Table 2).

**Table 2:** Baseline demographic and clinical characteristics of Medicaid beneficiaries newly prescribed HIV treatment

Baseline demographics and clinical characteristics	Adherence assessment population			Persistence assessment population		
	Overall patients (N = 1744)	STRs (N = 1290)	MTRs (N = 454)	Overall patients (N = 2409)	STRs (N = 1782)	MTRs (N = 627)
	N (%)	N (%)	N (%)	N (%)	N (%)	N (%)
Mean age (mean ± SD)	41.0 ± 12.9	40.0 ± 13.3	42.0 ± 11.8	40.1 ± 13.1	40.0 ± 13.5	41.0 ± 12.0
Age group (years)						
18–34	631 (36.2%)	493 (38.2%)	138 (30.4%)	918 (38.1%)	714 (40.1%)	204 (32.5%)
35–49	614 (35.2%)	432 (33.5%)	182 (40.1%)	805 (33.4%)	559 (31.4%)	246 (39.2%)
50–64	468 (26.8%)	338 (26.2%)	130 (26.8%)	646 (26.8%)	473 (26.5%)	173 (27.6%)
≥ 65	31 (1.8%)	27 (2.1%)	4 (0.9%)	40 (1.7%)	36 (2.0%)	4 (0.6%)
Gender						
Male	986 (56.5%)	741 (57.4%)	245 (54.0%)	1353 (56.2%)	1026 (57.6%)	327 (52.2%)
Female	758 (43.5%)	549 (42.6%)	209 (46.0%)	1056 (43.8%)	756 (42.4%)	300 (47.8%)
Race						
White	350 (20.1%)	270 (20.9%)	80 (17.6%)	472 (19.6%)	358 (20.1%)	114 (18.2%)
Black	1064 (61.0%)	779 (60.4%)	285 (62.8%)	1460 (60.6%)	1078 (60.5%)	382 (60.9%)
Hispanic	26 (1.5%)	18 (1.4%)	8 (1.8%)	37 (1.6%)	26 (1.5%)	11 (1.8%)
Other	304 (17.4%)	223 (17.3%)	81 (17.8%)	440 (18.3%)	320 (18.0%)	120 (19.1%)
Insurance type						
HMO	445 (25.5%)	342 (26.5%)	103 (22.7%)	635 (26.4%)	480 (26.9%)	155 (24.7%)
COMP	1298 (74.4%)	948 (73.5%)	350 (77.1%)	1773 (73.6%)	1302 (73.1%)	471 (75.1%)
Pre-index medication use						
Antihypertensive	439 (25.2%)	329 (25.5%)	110 (24.2%)	567 (23.5%)	425 (23.8%)	142 (22.6%)
Antidiabetics	106 (6.1%)	83 (6.4%)	23 (5.1%)	143 (5.9%)	107 (6.0%)	36 (5.7%)
Metformin	67 (3.8%)	54 (4.2%)	13 (2.9%)	86 (3.6%)	68 (3.8%)	18 (2.9%)
Metformin-combination	4 (0.2%)	3 (0.2%)	1 (0.2%)	5 (0.2%)	3 (0.2%)	2 (0.3%)
Non-insulin therapy	78 (4.5%)	63 (4.9%)	15 (3.3%)	101 (4.2%)	80 (4.5%)	21 (3.3%)
Insulin	43 (2.5%)	32 (2.5%)	11 (2.4%)	59 (2.5%)	43 (2.4%)	16 (2.6%)
Anticoagulants	102 (5.9%)	77 (6.0%)	25 (5.5%)	133 (5.5%)	101 (5.7%)	32 (5.1%)
Antiarrhythmic drugs	1 (0.1%)	1 (0.1%)	0 (0.0%)	1 (0.04%)	1 (0.1%)	0 (0.0%)
Lipid-lowering therapy	150 (8.6%)	111 (8.6%)	39 (8.6%)	193 (8.0%)	143 (8.0%)	50 (8.0%)
Statin	148 (8.5%)	110 (8.5%)	38 (8.4%)	191 (7.9%)	142 (8.0%)	49 (7.8%)
Ezetimibe	3 (0.2%)	2 (0.2%)	1 (0.2%)	3 (0.1%)	2 (0.1%)	1 (0.2%)
Statin/ezetimibe	1 (0.1%)	1 (0.1%)	0 (0.0%)	1 (0.04%)	1 (0.1%)	0 (0.0%)
PCSK9		0 (0.0%)	0 (0.0%)	0 (0.0%)	0 (0.0%)	1 (0.0%)
Antibiotics	759 (43.5%)	584 (45.3%)	175 (38.5%)	1068 (44.3%)	810 (45.5%)	258 (41.1%)
Respiratory drugs^a^	958 (54.9%)	735 (57.0%)	223 (49.1%)	1353 (56.2%)	1029 (57.7%)	324 (51.7%)
Number of unique medications on index date besides ART (mean ± SD)	1.0 ± 1.9	0.9 ± 1.8	1.3 ± 2.1	1.0 ± 2.0	1.0 ± 1.9	1.3 ± 2.3
Deyo-modified CCI score (mean ± SD)	4.2 ± 3.4	4.1 ± 3.4	4.4 ± 3.4	3.4 ± 3.5	3.4 ± 3.5	3.6 ± 3.5
Baseline clinical comorbidities						
Central nervous system toxicity	714 (40.9%)	530 (41.1%)	184 (40.5%)	811 (33.7%)	598 (33.6%)	213 (34.0%)
Gastrointestinal symptoms	74 (4.2%)	55 (4.3%)	19 (4.2%)	81 (3.7%)	60 (3.4%)	21 (3.3%)
Mental disorders	986 (56.5%)	732 (56.7%)	254 (55.9%)	1134 (47.1%)	835 (46.9%)	299 (47.7%)
AIDS-defining condition	118 (6.8%)	79 (6.1%)	39 (8.6%)	124 (5.2%)	82 (4.6%)	42 (6.7%)
Substance abuse	724 (41.5%)	522 (40.5%)	202 (44.5%)	827 (34.3%)	591 (33.2%)	236 (37.6%)
Jaundice	9 (0.5%)	6 (0.5%)	3 (0.7%)	9 (0.4%)	6 (0.3%)	3 (0.5%)
Dyslipidemia	251 (14.4%)	193 (15.0%)	58 (12.8%)	270 (11.2%)	209 (11.7%)	61 (9.7%)
Diabetes	149 (8.5%)	115 (8.9%)	34 (7.5%)	172 (7.1%)	131 (7.4%)	41 (6.5%)
Chronic kidney disease	58 (3.3%)	42 (3.3%)	16 (3.5%)	62 (2.6%)	46 (2.6%)	16 (2.6%)
Cardiovascular disease	562 (32.2%)	415 (32.2%)	147 (32.4%)	628 (26.1%)	465 (26.1%)	163 (26.0%)
Myocardial infarction	12 (0.7%)	8 (0.6%)	4 (0.9%)	12 (0.5%)	8 (0.4%)	4 (0.6%)

*STR* single-tablet regimen, *MTR* single-tablet regimen, *SD* standard deviation, *CCI* Charlson comorbidity index, *HMO* health maintenance organization, *COMP* comprehensive (patients were not incentivized to use a particular list of providers for non-emergency care, and coverage was handled by only one policy)

^a^Include drugs for lower and upper respiratory infections

### Treatment adherence

**Fig. 2:**
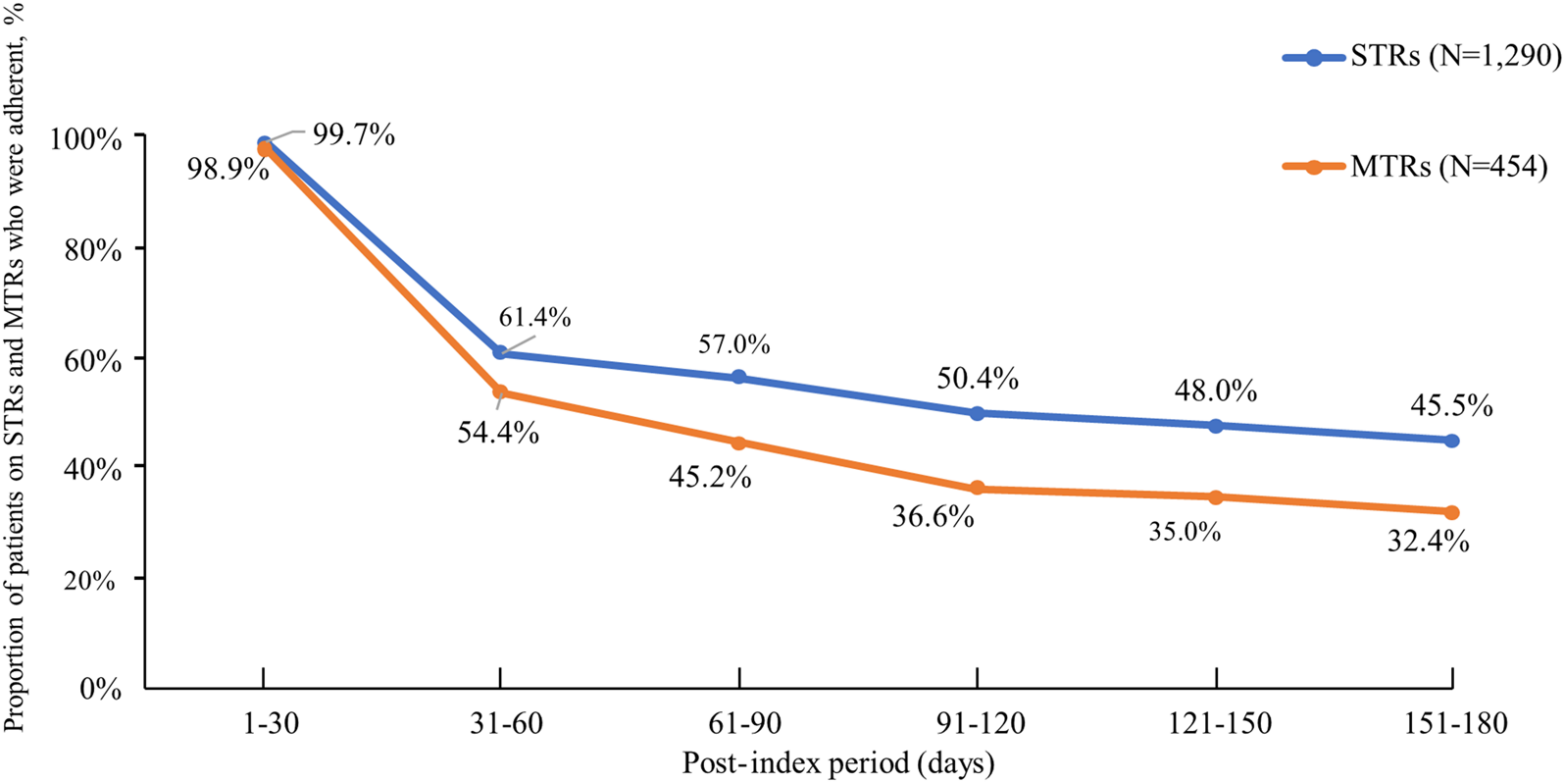
Proportion of adherent patients with newly prescribed HIV-1 treatment. *STR* single-tablet regimen, *MTR* multiple-tablet regimen; adherence: ≤ 5-day gap between successive fills (STRs), and ≤ 5-day gap between successive fills for one or more drugs in the regimen (MTRs)

Among patients who initiated STRs, nearly all the patients (99.7%) during the post-index period of 1-30 days were adherent, defined as a gap of ≤ 5 days between successive fills. However, adherence declined sharply by 38.4% during the post-index period of 31–60 days, corresponding to when patients would have had to refill a 30-day supply. Thereafter, a gradual decline was observed at each post-index period. Similarly, among patients who initiated MTRs, nearly all patients (98.9%) during the post-index period of 1–30 days were adherent, defined as having a gap of ≤ 5 days between successive fills for any drug in the regimen. Adherence declined by roughly 45.0% during the post-index period of 31–60 days, and decreased gradually at each post-index period afterwards. The proportion of adherent patients at each post-index period was higher among patients on STRs as compared to patients prescribed MTRs (Fig. 2). Likewise, during the post-index period of 1–180 days, the proportion of adherent patients was higher among patients who were prescribed STRs (22.7%) compared to MTRs (11.7%). Over 6-months of follow-up, 36.3% of patients initiating STRs and 48.8% of patients initiating MTRs discontinued therapy.

Among patients on STRs who were non-adherent, the mean refill gap at each post-index time interval ranged from 22–24 days with a cumulative mean refill gap of 71 days during the overall 6-month period. Likewise, among patients prescribed MTRs, the mean refill gap at each post-index time interval ranged from 18–25 days, with a cumulative mean refill gap of 81 days during the overall 6-month follow-up period. A greater proportion of patients prescribed MTRs had a cumulative refill gap of > 30 days as compared to STRs (MTRs: 65.6%; STRs: 53.0%).

When comparing those initiating STRs, the proportion of patients who were adherent during the post-index period of 1-180 days was highest among patients prescribed EVG/COBI/FTC/TAF (40.7%) followed by DTG/ABC/3TC (23.4%), EVG/COBI/FTC/TDF (22.4%), RPV/FTC/TDF (20.8%), and EFV/FTC/TDF (12.8%). When comparing patients who were non-adherent, the mean cumulative refill gap for those prescribed STRs was higher among those who initiated EFV/FTC/TDF (80 days) followed by RPV/FTC/TDF (76 days), EVG/COBI/FTC/TDF (70 days), ABC/3TC/DTG (66 days), and EVG/COBI/FTC/TAF (61 days) (Additional file 1: Figure S1).

Similarly, during the post-index period of 1-180 days, in the MTRs the proportion of adherent patients was highest among patients prescribed DTG + FTC/TDF (17.2%) followed by DRV/c + FTC/TDF (14.3%), ATV/c + FTC/TDF (12.8%), and DRV/r + FTC/TDF (5.4%). Furthermore, among patients taking DTG + FTC/TDF, adherence to the DTG component (25.8%) was more common than to the FTC/TDF component (18.5%). When comparing patients with MTR who were non-adherent, the mean cumulative refill gap was higher among those who initiated FTC/TDF + DRV/c (91 days), followed by FTC/TDF + ATV/c (86 days), FTC/TDF + DR/Vr (82 days), and FTC/TDF + DTG (76 days) (Additional file 2: Figure S2).

### Sensitivity analysis on STR adherence

Sensitivity analyses using an adherence definition of ≤ 7 gap days between successive fills yielded similar adherence rates as the primary definition of ≤ 5 days. Among the overall patients prescribed STRs, 28.0% were adherent and 72.0% were non-adherent during the post-index period of 1–180 days. By STR regimen type, patients prescribed EVG/COBI/FTC/TAF had the highest adherence during the post-index period of 1-180 days (46.7%). Likewise, findings based on sensitivity analyses using an adherence definition of ≤ 14 gap days between successive fills also yielded similar findings. Among the overall patients prescribed STRs, 39.8% were adherent during the post-index period of 1–180 days. By STR regimen type, patients prescribed EVG/COBI/FTC/TAF had the highest proportion of adherent patients during the post-index period of 1–180 days (58.5%).

### Treatment persistence

The median follow up time was 300 days (range: 4–730 days). Unadjusted analysis for persistence indicated that median time on treatment was significantly higher for patients prescribed STRs as compared to MTRs (166 vs 128 days; log-rank test p < 0.0001). Furthermore, 36.3% of STR patients discontinued the index regimen compared to 48.8% of MTR patients (Table 3). Comparing STRs, median treatment persistence was higher among patients treated with EVG/COBI/FTC/TDF (198 days), followed by DTG/ABC/3TC (180 days) and RPV/FTC/TDF (159 days). Because there was no minimum follow-up period required, some regimens, including EVG/COBI/FTC/TAF, RPV/FTC/TAF, and FTC/TAF, were not available during the entire study period, thereby limiting the 12-month persistence assessment. After controlling for baseline differences, discontinuation of the first-line therapy was highest for patients treated with EFV/FTC/TDF (51.0%) followed by EVG/COBI/FTC/TDF (47.2%) and RPV/FTC/TDF (46.9%). The proportion of patients discontinuing first-line STR therapy was lowest for EVG/COBI/FTC/TAF (11.7%). When comparing MTRs, median treatment persistence was higher among patients treated with FTC/TDF + DTG (151 days) followed by ABC/3TC + DRV/r (146 days) and FTC/TDF + DRV/c (144 days). First-line therapy discontinuation was highest for patients treated with FTC/TDF + DRV/r (59.8%), followed by FTC/TDF + ATV/r (58.7%) and FTC/TDF + DRV/c (50.0%). The proportion of patients discontinuing first-line MTR therapy was lowest for ABC/3TC + DRV/r (25%), albeit among just 12 patients (Table 3).

**Table 3:** Persistence on index treatment among Medicaid beneficiaries newly prescribed ART

Persistence assessment	Number of days on therapy	Patients with 12-month persistence among those with 12 months follow-up	Patients with discontinuation of first-line therapy^b^
	Median	N (%)	N (%)
All Regimens (STR + MTR) [N = 2409]	156.0	410 (43.2%)	953 (39.6%)
STRs [N = 1782]	166.0	313 (45.0%)	647 (36.31%)
EVG/COBI/FTC/TAF [N = 274]	145.0	8 (100.0%)^†^	32 (11.7%)
EVG/COBI/FTC/TDF [N = 475]	198.0	111 (43.4%)	224 (47.2%)
ABC/3TC/DTG [N = 522]	180.0	106 (53.3%)	152 (21.6%)
RPV/FTC/TAF [N = 27]	131.0	N/A^a^	0 (0.0%)
RPV/FTC/TDF [N = 196]	158.5	37 (38.9%)	92 (46.9%)
EFV/FTC/TDF [N = 288]	140.0	51 (37.2%)	147 (51.0%)
MTRs [N = 627]	128.0	97 (38.0%)	306 (48.8%)
ABC/3TC + DRV/r [N = 12]	146.0	2 (50.0%)	3 (25.0%)
FTC/TDF + DTG [N = 205]	151.0	32 (47.8%)	92 (44.9%)
FTC/TAF + DTG [N = 31]	79.0	N/A^a^	0 (0.0%)
FTC/TDF + DRV/r [N = 184]	119.0	34 (33.3%)	110 (59.8%)
FTC/TDF + DRV/c [N = 50]	144.0	4 (30.8%)	25 (50.0%)
FTC/TDF + ATV/r [N = 121]	120.0	25 (37.3%)	71 (58.7%)

MTRs including ABC/3TC + DRV/c, FTC/TAF/r/c, FTC/TDF + ATV/c, and FTC/TAF + ATV/r/c were not examined due to the limited sample size

*3TC* lamivudine, *ABC* abacavir, *ATV* atazanavir, *ATV/c* atazanavir boosted with cobicistat, *ATV/r* atazanavir boosted with ritonavir, *COBI* cobicistat, *DRV* darunavir, *DRV/r* darunavir boosted with ritonavir, *DRV/c* darunavir boosted with cobicistat, *DTG* dolutegravir, *EFV* efavirenz, *EVG* elvitegravir, *FTC* emtricitabine, *MTR* multi-tablet regimen, *RPV* rilpivirine, *STR* single tablet regimen, *TAF* tenofovir alafenamide fumarate, *TDF* tenofovir disoproxil fumarate

^a^EVG/COBI/FTC/TAF was approved by the FDA in November 2015, RPV/FTC/TAF was approved March 2016, and FTC/TAF was approved April 2016, thus limiting the number of patients with 12 months of follow-up

^b^Age groups (18–34 years, 35–49 years, 50–64 years and ≥ 65 years), gender (female and male), race (White, Black, Hispanic and other), insurance type (HMO and COMP), baseline clinical comorbidities (central nervous system toxicity, gastrointestinal symptoms, mental disorders, AIDS-defining condition, substance abuse, jaundice, dyslipidemia, diabetes, chronic kidney disease, cardiovascular disease and myocardial infarction), pre index medication use (antihypertensive, antidiabetics, anticoagulants, antiarrhythmic drugs, lipid-lowering therapy, antibiotics and respiratory drugs), number of unique medications on index date other than antiretroviral therapy (ART), and Deyo-modified CCI score

Furthermore, findings from the multivariable Cox proportional hazard model indicated that after controlling for the baseline differences, patients with MTR had a higher risk of treatment discontinuation (HR: 1.6; 95% confidence interval [CI] 1.3–1.8; p < 0.0001). Similarly, comparing STR patients, persistence was significantly higher for those treated with EVG/COBI/FTC/TAF as compared to EFV/FTC/TDF (HR: 3.6; 95% CI 2.4–5.3; p < 0.0001), RPV/FTC/TDF (HR: 3.1; 95% CI 2.0–4.6; p < 0.0001), EVG/COBI/FTC/TDF (HR: 2.8; 95% CI 1.9–4.1; p < 0.0001), and DTG/ABC/3TC (HR: 1.8; 95% CI 1.2–2.6; p = 0.004).

**Fig. 3:**
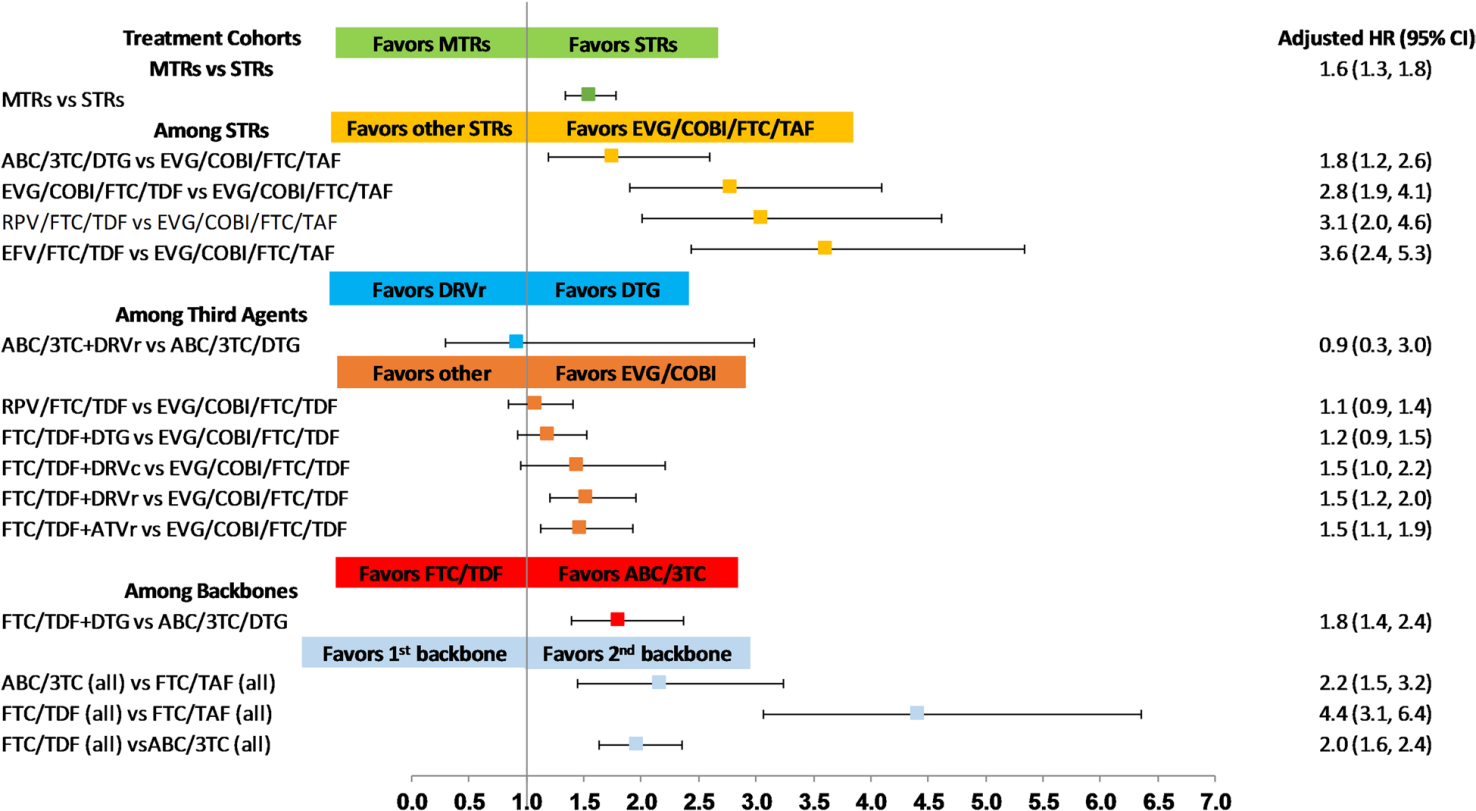
Adjusted hazard ratios for risk of treatment discontinuation among treatment cohorts. *STR* single-tablet regimen, *MTR* multiple-tablet regimen, *3TC* lamivudine, *ABC* abacavir, *ATV* atazanavir, *ATV/c* atazanavir boosted with cobicistat, *ATV/r* atazanavir boosted with ritonavir, *COBI* cobicistat, *DRV* darunavir, *DRV/r* darunavir boosted with ritonavir, *DRV/c* darunavir boosted with cobicistat, *DTG* dolutegravir, *EFV* efavirenz, *EVG* elvitegravir, *FTC* emtricitabine, *RPV* rilpivirine, *TAF* tenofovir alafenamide fumarate, *TDF* tenofovir disoproxil fumarate. ABC/3TC (all), FTC/TAF (all), and FTC/TDF (all) are backbones of STRs and MTRs compared, regardless of their third agents

Comparing third agents, persistence was significantly higher for patients with EVG/COBI use as compared to DRV/r (HR: 1.5; 95% CI 1.2–2.0; p = 0.001) and ATV/r (HR: 1.5; 95% CI 1.1–1.9; p = 0.006) (Fig. 3). Furthermore, when comparing backbones, persistence was significantly higher for patients taking an FTC/TAF-based regimen as compared to an ABC/3TC- (HR: 2.2; 95% CI 1.5–3.2; p < 0.0001) or FTC/TDF-based regimen (HR: 4.4; 95% CI 3.1–6.4; p < 0.0001), regardless of the third agent. Similarly, the ABC/3TC backbone was associated with higher persistence compared to FTC/TDF (HR: 2.0; 95% CI 1.6–2.4; p < 0.0001), regardless of the third agent.

## Discussion

The discontinuation caused by poor tolerability, poor adherence, or complexity carries risks of the development of toxicity and resistance associated with compromised future treatment options. Improved rates of adherence and persistence are critically important for the long-term therapy of HIV patients [32] and improved health outcomes. In this retrospective claims-based study including several thousand Medicaid recipients newly initiating ART, treatment adherence and persistence were examined and compared among patients prescribed STRs and MTRs.

ART adherence has been shown to improve health outcomes and QoL, including HIV transmission, viral load suppression, drug resistance prevention, and survival rates [4]. Most research examining adherence to ART defines adherence based on either medication possession ratio (MPR) or proportion of days covered (PDC) [27, 33–35]. MPR and PDC are the conventional measure of adherence but are not necessarily the gold standard. The current study’s approach of defining adherence as a < 5 day gap in fills over 30-day intervals during the 6-month follow-up period is more stringent in allowing for examination of the proportion of patients who are adherent and non-adherent at different time points from therapy initiation date. The current study found that during the overall 6-month follow-up period, the proportion of adherent patients was higher among STR initiators (22.7%) compared to MTR initiators (11.7%); this is similar to the finding from Hines et al., where the same measure of adherence was applied and the proportion of adherent patients for STR initiators and MTR initiators were 24.9% and 11.7%, respectively [36]. In support of our study, an observational study of 755 people living with HIV recruited from community services found that patients taking STRs were more likely to adhere to their medication compared to those on MTRs [5]. Lowering pill burden is associated with improved adherence and viral suppression, and consequently QoL [4, 37, 38]. In further support of our study findings Sutton et al. showed that an STR cohort had significantly better adherence when compared to an MTR cohort; additionally, risk of hospitalization was lower in the STR cohort [33]. Although our study did not examine barriers to adherence, a study by Chen et al. reported that MTR patients were more likely to report barriers including scheduling, side-effects, and confusion over their prescription as reasons for missing a dose compared to those prescribed STRs [5]. Comparing the STRs, the adherence was highest among those prescribed EVG/COBI/FTC/TAF in contrast to all other STRs.

Adherence and persistence are also considered cornerstones of effective ART therapy; hence, it is recommended that ART regimens should be efficacious, with no virologic failures, well-tolerated, and without toxicity, to improve adherence, and, in turn, improve long-term persistence [39, 40]. The unadjusted analysis results in the current study show that a lower proportion of STR patients discontinued their first-line therapy (36.3%) compared to MTR patients (48.8%). Likewise, the adjusted analysis revealed that MTR patients had a 1.6 times greater hazard of treatment discontinuation as compared to STR patients. In support of this finding, a recent claims-based study among Medicaid beneficiaries also revealed that persistence is greater with less toxic ART regimens that include fewer pills. The study further reported that there was a 29% reduced hazard of non-persistence with STRs compared with regimens containing > 6 ART pills per day [41]. Another study also reported that STR discontinuation was lower (i.e., few patients discontinued the STR throughout the study), and further found that patients who switched from an MTR to an STR were able to maintain virologic suppression [14]. Although outside the scope of the current analysis, future research should investigate reasons for discontinuation and switching of therapy. Past research has reported that factors including pill burden, poor tolerability, risk of resistance due to complexity, toxicity, regimen performance, drug efficacy, and virologic failure could be possible reasons for higher rates of discontinuation among MTR patients [20, 37, 40]. Furthermore, the current study also showed that among STRs, patients who were treated with EVG/COBI/FTC/TAF had greater persistence compared to those treated with other STRs. Since EVG/COBI/FTC/TAF has been found to have higher efficacy due to fewer drug-related adverse events compared to EVG/COBI/FTC/TDF among treatment naïve patients [42], providers presumably switched regimens based on those data as well as changes to HIV treatment guidelines.

The current study examined regimen components in addition to specific STRs and MTRs. Separate comparisons were performed among patients prescribed third agents and backbones to explicitly explore the benefits derived from each individual component. Our study found that patients who were treated with regimens including an FTC/TAF backbone had higher persistence compared to regimens with ABC/3TC and FTC/TDF, providing one of the first real world studies assessing persistence of the FTC/TAF backbone, which was approved in 2016. While reasons for persistence could not be assessed in this analysis, the higher persistence of FTC/TAF likely relates to clinical trial data showing high efficacy and bone and renal safety advantages compared to prior regimens, and the 2018 IAS-USA guidelines recommended TAF but not TDF as a component of initial suggested regimens [43, 44].

The findings from our study should be viewed in the context of study and claims data limitations. While claims data are extremely valuable for the efficient and effective examination of health care outcomes, they are primarily collected for business purposes rather than research purposes. Therefore, analyses may be subject to inherent limitations of the source administrative claims data, such as coding errors or diagnoses entered for administrative processing rather than clinical completeness. Moreover, the presence of a claim for a filled prescription does not indicate that the medication was consumed or taken as prescribed. Also, medications filled over-the-counter or provided as samples by the physician are not observed in claims data. Certain information that could influence study outcomes is not readily available in claims data, such as clinical and disease-specific parameters including HIV viral load, which could be used as a surrogate for adherence. In addition, although the study adjusted for observed baseline characteristics in the multivariable analyses, some unobserved confounders may remain. Further, given the study timeframe, newly-available HIV regimens were not included. Lastly, this study was limited in that health claims may be subject to error; however, given that ART is only used for HIV and that at least two ART claims were required for inclusion, it is reasonable to conclude that patients identified in the Truven Medicaid population by ART use are HIV patients.

## Conclusions

In this analysis of real world claims data associated with US Medicaid recipients newly initiating ART, adherence and persistence was greater among patients initiating STRs than those initiating MTRs. In fact, the proportion of patients who were adherent was almost double for those initiating STRs compared to MTRs. Additionally, median treatment persistence was also higher among STR initiators. Regimens containing EVG/COBI as a third agent and FTC/TAF as a backbone showed higher persistence than other third agents and backbones, respectively. In addition, both adherence and persistence were greater with single-tablet EVG/COBI/FTC/TAF than with other STRs. As new regimens with improved safety and tolerability profiles become available, continued research on persistence and adherence for HIV treatments is warranted to inform better therapeutic management.

## Supplementary information

**Additional file 1: Figure S1.** Proportion of Adherent Patients on Individual STRs. *3TC* Lamivudine, *ABC* Abacavir, *COBI* Cobicistat, *DTG* Dolutegravir, *EVG* Elvitegravir, *FTC* Emtricitabine, *RPV* Rilpivirine, *STR* single tablet regimen, *TAF* Tenofovir Alafenamide Fumarate, *TDF* Tenofovir Disoproxil Fumarate; Adherence ≤ 5-day gap between successive fills.

**Additional file 2: Figure S2.** Proportion of Adherent Patients on Individual MTRs. *ATV/c* Atazanavir boosted with cobicistat, *DRV/c* Darunavir boosted with cobicistat, *DRV/r* Darunavir boosted with ritonavir, *DTG* Dolutegravir, *FTC* Emtricitabine, *TDF* Tenofovir Disoproxil Fumarate; Adherence: ≤ 5 days gap in fill for one or more drugs in the regimen.

## Data Availability

The datasets used and/or analyzed during the current study are available from the corresponding author on reasonable request.
