# Supplementary figures and images for: Real-world adherence and persistence for newly-prescribed HIV treatment: single versus multiple tablet regimen comparison among US medicaid beneficiaries
Source: AIDS Res Ther. 2020 Apr 1;17:12. doi: 10.1186/s12981-020-00268-1 (PMC7110826; doi:10.1186/s12981-020-00268-1)

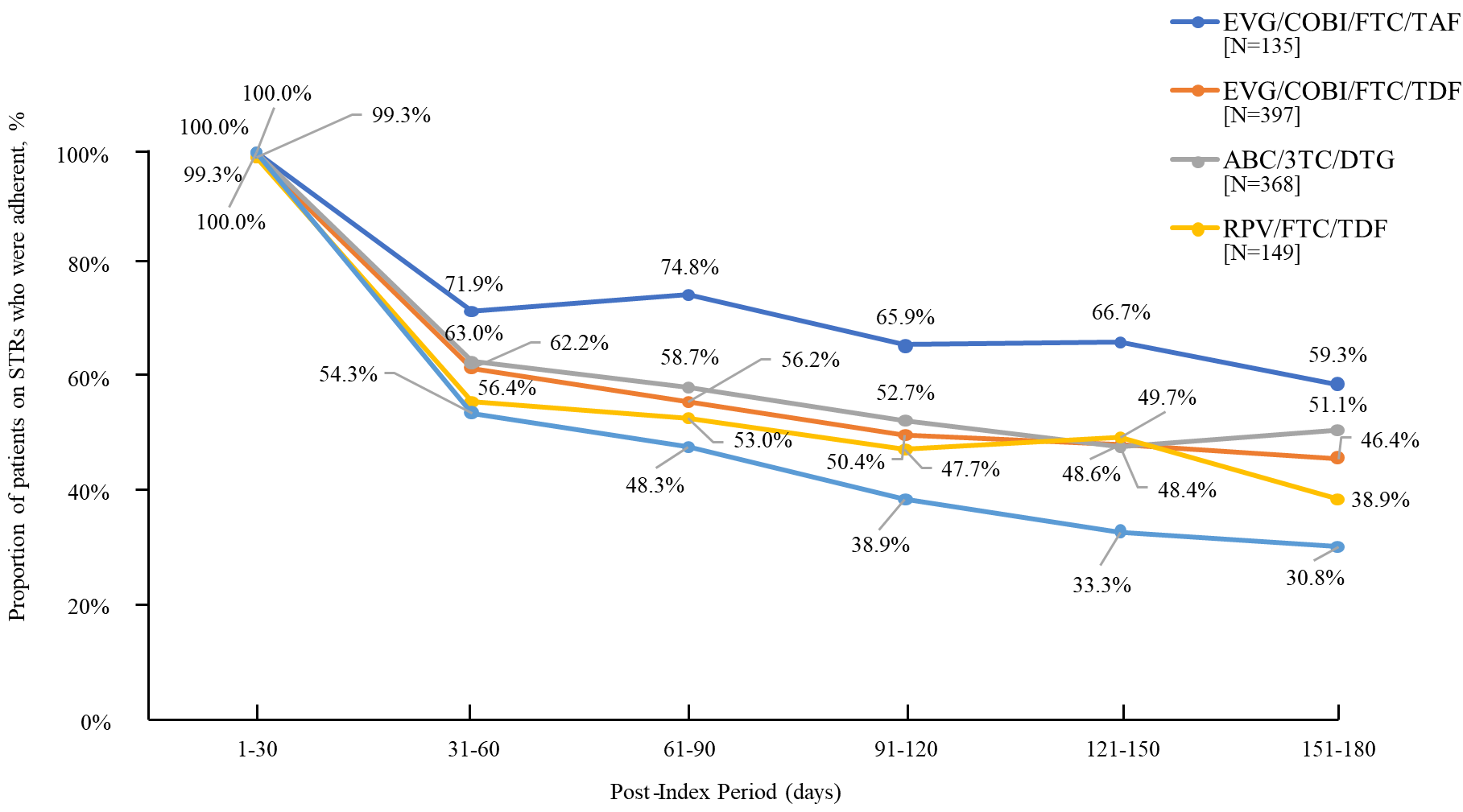

Supplement: Supplementary file 1 — Additional file 1: Figure S1. Proportion of Adherent Patients on Individual STRs. 3TC Lamivudine, ABC Abacavir, COBI Cobicistat, DTG Dolutegravir, EVG Elvitegravir, FTC Emtricitabine, RPV Rilpivirine, STR single tablet regimen, TAF Tenofovir Alafenamide Fumarate, TDF Tenofovir Disoproxil Fumarate; Adherence ≤ 5-day gap between successive fills. [file 12981_2020_268_MOESM1_ESM.tif]

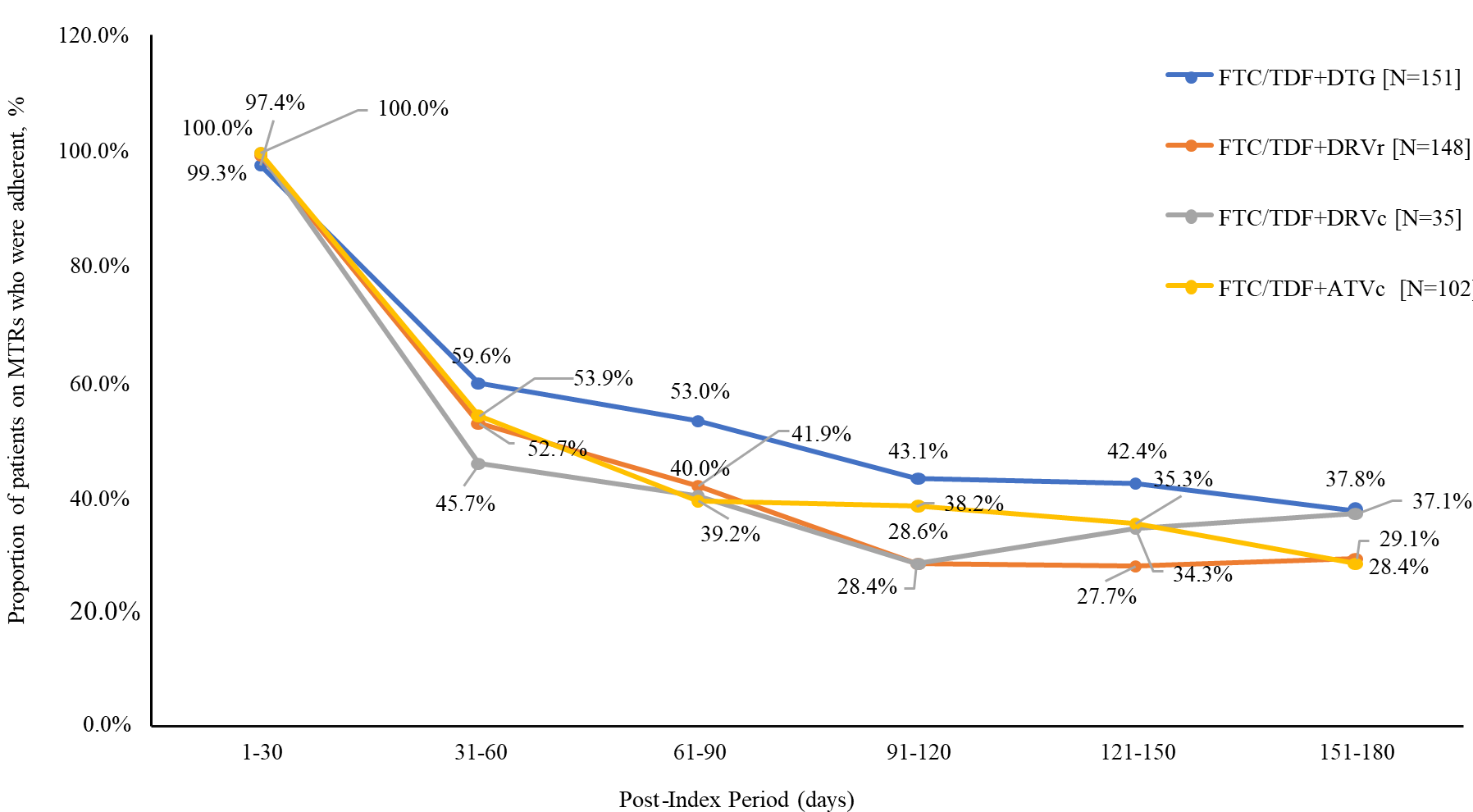

Supplement: Supplementary file 2 — Additional file 2: Figure S2. Proportion of Adherent Patients on Individual MTRs. ATV/c Atazanavir boosted with cobicistat, DRV/c Darunavir boosted with cobicistat, DRV/r Darunavir boosted with ritonavir, DTG Dolutegravir, FTC Emtricitabine, TDF Tenofovir Disoproxil Fumarate; Adherence: ≤ 5 days gap in fill for one or more drugs in the regimen. [file 12981_2020_268_MOESM2_ESM.tif]
